# MicroRNA-155 mediates multiple gene regulations pertinent to the role of human adipose-derived mesenchymal stem cells in skin regeneration

**DOI:** 10.3389/fbioe.2024.1328504

**Published:** 2024-03-18

**Authors:** Hady Shahin, Luigi Belcastro, Jyotirmoy Das, Marina Perdiki Grigoriadi, Rolf B. Saager, Ingrid Steinvall, Folke Sjöberg, Pia Olofsson, Moustafa Elmasry, Ahmed T. El-Serafi

**Affiliations:** ^1^ Department of Hand Surgery, Plastic Surgery, and Burns, Linkoping University Hospital, Linköping, Sweden; ^2^ Department of Biomedical and Clinical Sciences, Linkoping University, Linköping, Sweden; ^3^ Faculty of Biotechnology, Modern Sciences and Arts University, October City, Cairo, Egypt; ^4^ Department of Biomedical Engineering, Linkoping University, Linköping, Sweden; ^5^ Bioinformatics Unit, Core Facility (KEF), Faculty of Medicine and Health Sciences (BKV), Linköping University, Linköping, Sweden; ^6^ Clinical Genomics Linköping, SciLife Laboratory, Department of Biomedical and Clinical Sciences, Linköping University, Linköping, Sweden; ^7^ Department of Clinical Pathology, Linkoping University Hospital, Linköping, Sweden

**Keywords:** adipose-derived mesenchymal stem cells, miRNA, miR-155-5p, proteome, porcine wound model, wound healing, fibroblast growth factors

## Abstract

**Introduction:** The role of Adipose-derived mesenchymal stem cells (AD-MSCs) in skin wound healing remains to be fully characterized. This study aims to evaluate the regenerative potential of autologous AD-MSCs in a non-healing porcine wound model, in addition to elucidate key miRNA-mediated epigenetic regulations that underlie the regenerative potential of AD-MSCs in wounds.

**Methods:** The regenerative potential of autologous AD-MSCs was evaluated in porcine model using histopathology and spatial frequency domain imaging. Then, the correlations between miRNAs and proteins of AD-MSCs were evaluated using an integration analysis in primary human AD-MSCs in comparison to primary human keratinocytes. Transfection study of AD-MSCs was conducted to validate the bioinformatics data.

**Results:** Autologous porcine AD-MSCs improved wound epithelialization and skin properties in comparison to control wounds. We identified 26 proteins upregulated in human AD-MSCs, including growth and angiogenic factors, chemokines and inflammatory cytokines. Pathway enrichment analysis highlighted cell signalling-associated pathways and immunomodulatory pathways. miRNA-target modelling revealed regulations related to genes encoding for 16 upregulated proteins. miR-155-5p was predicted to regulate Fibroblast growth factor 2 and 7, C-C motif chemokine ligand 2 and Vascular cell adhesion molecule 1. Transfecting human AD-MSCs cell line with anti-miR-155 showed transient gene silencing of the four proteins at 24 h post-transfection.

**Discussion:** This study proposes a positive miR-155-mediated gene regulation of key factors involved in wound healing. The study represents a promising approach for miRNA-based and cell-free regenerative treatment for difficult-to-heal wounds. The therapeutic potential of miR-155 and its identified targets should be further explored *in-vivo*.

## 1 Introduction

Adipose-derived mesenchymal stem cells (AD-MSCs) are known for their plasticity and low immunogenicity, with adequate yield isolated from fat biopsies and lipoaspirate (Gimble and Guilak, 2003; Kern et al., 2006; Bailey et al., 2010). Upon appropriate stimulation, AD-MSCs have the potential to differentiate into a wide variety of cell types, including osteoblasts, chondrocytes, myocytes, adipocytes, fibroblasts, smooth muscle, endothelial and epithelial cells (Gimble et al., 2007; Hassan et al., 2014; Azari et al., 2022). When applied *in-vivo*, AD-MSCs can enhance tissue regeneration through their differentiation into skin cells or secretion of paracrine factors. The latter can initiate the healing process via recruiting circulating stem cells, as well as other local cells to the wound microenvironment. AD-MSCs have shown potential to differentiate into various skin cell types including fibroblasts and keratinocytes. In addition, AD-MSCs can promote wound healing by releasing growth factors and cytokines, which promote epithelial migration, neovascularization and collagen synthesis (Hassan et al., 2014). AD-MSCs modulate the immune response by suppressing T-cell proliferation and inducing the production of regulatory T-cells, which play a critical role in supressing the inflammatory response and promotion of tissue regeneration (Lin et al., 2013; Ong et al., 2017; Weiss and Dahlke, 2019). These features rendered AD-MSCs a viable alternative to epidermal cells in skin regenerative applications, particularly in cases of extensive skin loss as the donor sites for keratinocyte isolation are scarce (Gimble et al., 2007; Cherubino et al., 2011; Kokai et al., 2014; Kirby et al., 2015; Foubert et al., 2016; Bertozzi et al., 2017).

In this study, we investigated the effect of AD-MSCs in a porcine full thickness skin wound model and the healing was evaluated using histopathology and spatial frequency domain imaging (SFDI). SFDI is an imaging technique that projects a sequence of sinusoidal patterns of illumination with varying spatial frequencies onto tissue and utilizes a camera to detect diffusely remitted light. By measuring both the reduction of remitted light intensity and contrast from these patterns, models of light transport can calculate the amount of absorption and light scattering present within the tissue. Another aspect of interest in SFDI is the penetration depth of light, which depends on the spatial frequency (f_x_) of the sinusoidal patterns (O’Sullivan et al., 2012). Quantifying these absorption and scattering properties over multiple wavelengths permits physiologic interpretations of these optical signals (O’Sullivan et al., 2012; Zannettino et al., 2008; Yafi et al., 2011; Saager et al., 2013; Murphy et al., 2020; Lee et al., 2020; Ponticorvo et al., 2020). This technique has been previously applied to burn wound assessment (Ponticorvo et al., 2019; Ponticorvo et al., 2020), chronic wounds (Lee et al., 2020; Murphy et al., 2020), and several other clinical applications (Yafi et al., 2011; Saager et al., 2013). To our knowledge, this is the first application of this imaging approach to assess cell-based therapeutics in a surgical wound model.

The general understanding regarding AD-MSCs’ mechanism of wound repair, underlying pathways and interplay with other cell types is currently insufficient. At the wound site, AD-MSCs interact with surrounding tissue, which is crucial to achieve skin repair. microRNA (miRNA), fragments of short non-coding RNA, can be considered as one of the most important mediators in cell-to-cell communication. miRNA can disseminate through the extracellular fluid to act as signaling molecules or through direct exchange of exosomes between adjacent cells. miRNA can also shuttle as a cargo in the exosomes, locally and through the systemic circulation. The ultimate effect is the alteration of gene expression and protein production in the recipient cell (Alghfeli et al., 2022). miRNA are involved in the post-transcriptional regulation of protein-coding genes by interfering with messenger RNA transcript, leading to its degradation, or -at least-repression of protein production (Collins, 2011). miRNA can influence diverse biological processes including cell growth, development, metabolism, migration, proliferation, differentiation and apoptosis (Kim and Sung, 2017; Omar et al., 2019). Additionally, miRNA have been shown to regulate various aspects of wound healing including cell proliferation, migration, collagen biosynthesis, and angiogenesis (Wang et al., 2012; Banerjee and Sen, 2013; Veith et al., 2019). On the other hand, proteins released by AD-MSCs act as stimulants for cell growth, tissue granulation, increased macrophage recruitment and improved neovascularization. These proteins include growth factors, cytokines and chemokines, such as vascular endothelial growth factor (VEGF), insulin-like growth factor (IGF), and transforming growth factor-beta (TGF-β), among others (El-Serafi et al., 2017; Ong et al., 2017; Shahin et al., 2020; Azari et al., 2022).

AD-MSCs treated wounds showed better properties of healing that mimicked natural healing. We performed an integrated analysis of our previously published miRNome data in primary human AD-MSCs, as well as to relevant protein content, in comparison to normal keratinocytes as the primary effector in physiological cutaneous wound edge healing (Shahin et al., 2023). The findings were validated through gene expression study and via anti-miR-driven experimental targeting. The aim of the study was to identify the main factors involved in skin wound repair produced by AD-MSCs through a miRNA-based approach.

## 2 Materials and methods

### 2.1 Experimental animal procedures

All animal procedures were carried out according to the animal research ethical approval (No. 1961 DNR 5.2.18-1627/15) in compliance with the guidelines mandated by the regional animal ethics review board and the Animal Research: Reporting of *In Vivo* Experiments (ARRIVE) guidelines to ensure humane treatment of research animals. Two female pigs (*Sus scrofa domesticus*) weighing between 50 and 60 kg were obtained from a licensed breeder and included in this study. Animals were housed in enclosures measuring 2 × 2.5 m^2^, with a light/dark cycle of 12 h/12 h and an ambient temperature of 18°C–20°C. The animals had free access to water and hay and were fed twice daily. Surgical procedures were performed under general anaesthesia, induced by intramuscular injection of 10 μg/kg dexmedetomidin (Dexdomitor; OrionPharma, Danderyd, Sweden) and 3 μg/kg of tiletamin and zolazepam (Zoletil; Virbac, Kolding, Denmark). Animals were intubated with an endotracheal tube connected to an automatic ventilator. General anaesthesia and analgesia were maintained with intravenous infusion of 3–7.5 mg/kg pentobarbital sodium (Pentobarbitalnatrium vet.; APL, Kungens Kurva, Sweden) in combination with 0.5−0.75 μg/kg fentanyl (Leptanal, Janssen, Solna, Sweden). Vital parameters were monitored by pulse oximetry, capnography and rectal thermometer. Signs of postoperative pain were treated with fentanyl patches, 25–50 μg/h for 72 h (Fentanyl Orion; Orion Pharma, Danderyd, Sweden).

### 2.2 Porcine wound model

For cell isolation, two full-thickness skin biopsies, measuring 3–5 cm^2^, were collected from the neck region of each animal. The wounds were closed with sutures with prolene 2–0 (Ethicon Inc.; Somerville, New Jersey, United States) and covered with sterile gauze. Porcine AD-MSCs (*p*AD-MSCs) were extracted from the subcutaneous adipose layer after mechanical separation from the dermis layer. Briefly, 20 mL of adipose tissue were cut into 0.5–1 cm^2^ slices and incubated with Collagenase I (1 mg/mL) (Gibco, Life Technologies, United Kingdom) at a 3:1 volume ratio on a tube rotator at 37°C for 90 min. The reaction was stopped by adding Dulbecco’s Modified Eagle Medium (Gibco, Billings, MT, United States) with 10% fetal bovine serum (Life Technologies, São Paulo, Brazil). The cells were centrifuged and washed, at least twice, with phosphate buffered saline (PBS; Life Technologies, Grand Island, NY, United States) then suspended in 2.5% human serum albumin (HSA) (Alburex, CSL Behring GmbH, Marburg, Germany). The following day the animals (*n* = 2) were anesthetized and sixteen full-thickness excisional wounds with a radius of 1.5 cm to resemble non-healing wounds were created paravertebrally on the dorsum of each animal. The experimental group of *p*AD-MSCs and a negative control were randomly assigned to a wound on each pig. Wounds were covered with occlusive dressings (Tegaderm™, 3M™, MN, United States). Then 1 × 10^6^ *p*AD-MSCs cell suspension was injected through the dressing into the wound cavity. The injection site was sealed with another layer of the dressing. The control wounds were left untreated to delineate physiological healing. An absorbent foam dressing (Mepilex XT, Mölnycke, Sweden) was added over the Tegaderm and finally the whole trunk was wrapped with an elastic dressing (Elastic bandage, Hansbo sport, Västra Frölunda, Sweden). At 0, 7 and 14-day time points, planimetric measurements (wound diameters) were taken for each wound. The progress of wound healing was clinically evaluated by a plastic surgeon at day 7 and 14. At the 2-week mark, the animals were euthanized by intravenous injection of 400 mg/kg pentobarbital sodium (Pentobarbitalnatrium vet.; APL, Kungens Kurva, Sweden).

### 2.3 Wound evaluation using spatial frequency domain imaging

A wide-field spectral imaging system was employed to non-invasively quantify the changes within the wound areas at 0, 7, and 14-day time-points. The specific imaging system is a compact, low-cost, custom-built imager, developed at Linkoping University (Belcastro et al., 2020). The system is capable of imaging tissue in a field of view of approximately 5 cm width, over five spectral bands (458, 520, 536, 556, 626 nm). 11 spatial frequencies were acquired, ranging from 0 to 0.5 mm^−1^, in steps of 0.05 mm^−1^. Patterns with lower f_x_ penetrate deeper in the tissue, while higher f_x_ is shallower. Making use of this property, the dataset of 11 f_x_ was subdivided into smaller subsets containing 4 frequencies each. These subsets contain information about different volumes, which allow comparative measurements from deeper and more superficial tissue (O’Sullivan et al., 2012) and make qualitative interpretations on the state of new tissue growth and the underlying supporting structures. Since the data is in image format, a rectangular area was drawn over the wound sites as a region of interest. The mean and standard deviation of the parameters area were calculated at each pixel, to account for the spatial heterogeneity of the wound, for at least 200 pixels/wound. The mean scattering coefficient (µ’s) was then fit to the scattering parameters A and B. The dependance of µ’s to the wavelength of light (λ) can be modelled with a power law as $\mu_{s}^{\prime }\left(\lambda \right)=A\lambda^{-B}$ , where the two scattering parameters amplitude (A) and slope (B) are related respectively to the concentration of scattering particles and their average dimension (Mourant et al., 1998). Higher parameter A reflects greater number of scattering particles while higher B parameter indicates smaller size of these particles. Objects that scatter light in tissue, range from “large” nuclei to “small” collagen fibrils and other extracellular matrix (ECM) components, so the B parameter can be used as a rough estimator of the most common structures in the tissue. In the context of wound healing, increases in both A and B parameters represent new cell growth (re-epithelialization) and collagen restructuring/formation. In this study, the SFDI data in two spatial frequency ranges (f1 and f2) were processed. These distinct ranges provide insight into the relative depths at which these changes in tissue structure (scattering) and function (absorption) occur. Data from f1 represents deeper tissue volumes, ranging from tissue surface to approximately 0.3–0.5 mm deep, and f2 is restricted to the more superficial tissue (surface to ∼0.25–0.35 mm).

### 2.4 Tissue preparation and histological examination

Biopsies were obtained at day 14. The wounds were excised using a scalpel with an approximately 3 mm margin. Biopsies were fixed in 4% buffered formaldehyde, dehydrated by immersion in a series of ethanol-xylene and embedded in paraffin. Tissue sections were cut into 4–5 µm thickness, deparaffinized in xylene, rehydrated and stained with hematoxylin and eosin (H&E) as well as Masson’s trichrome stain (Sigma Aldrich, United States). The slides were evaluated by a specialist skin pathologist blinded to the study design and images were captured using the Imageview software version X64 (Olympus Corp., Japan). The epidermal thickness was measured using the analysis function of Adobe Photoshop 2023 with measurement scale customized to the scale bar. At least, twenty readings were obtained for each group.

### 2.5 Analysis of miRNA microarray and protein array data and pathway enrichment

The miRNA and protein screening data in Keratinocytes and AD-MSCs (Shahin et al., 2023), was analysed to identify the upregulated proteins in AD-MSCs. Our reported dataset “Differentially expressed DEmiRNA-mRNA Interaction Network analysis” in AD-MSCs versus keratinocytes was utilized to identify the enriched pathways in AD-MSCs. From this analysis, 555 unique target genes related to 58 upregulated miRNA in AD-MSCs were used as input list#1 in the Reactome database (v83, https://reactome.org/) (Yu and He, 2016) for pathway enrichment analysis. Similarly, genes encoding upregulated proteins in AD-MSCs were also investigated independently for pathway enrichment as input list#2. The following filters were applied, 1) species: *Homo sapiens*, 2) statistical significance: *p* < 0.05. The resulting lists of pathways from each analysis were compared to highlight the top matching enriched pathways.

### 2.6 Predictive miRNA-mediated gene regulations (integrated analysis)

The two-tailed Fisher’s exact test (2 × 2 contingency table) was used to test the null-hypothesis of no association between DEmiRNA and differentially expressed proteins. Integrated analysis was performed to extract predictive miRNA-mediated gene regulations in AD-MSCs. To maintain a reliable source for miRNA target genes, the Affymetrix GeneChip^®^ miRNA 4.0 array annotation (v. HG38) containing the experimentally validated targets gene symbols corresponding to miRNA using miRecords for validated targets. All possible gene names corresponding to the genes encoding for the experimentally identified upregulated proteins in AD-MSCs were crossed matched with the annotated gene targets for all differentially expressed miRNA. The matching DEmiRNA with target genes encoding for any of the upregulated proteins in AD-MSCs were identified and categorized according to the following 2 assumed regulations:

$$\begin{array}{c} miRNA\;\to \;Protein-coding\;gene\;\to \;Protein\\ OR\\ miRNA\;─┤\;Protein-coding\;gene\;\to \;Protein \end{array}$$

### 2.7 Transfection with miR-155-5p inhibitor

ASC52telo, hTERT immortalized human adipose-derived mesenchymal stem cells (ATCC, United States) were used as a model for AD-MSCs in the transfection study. Lipofectamine RNAiMax (Thermo Fisher Scientific, Waltham, MA, United States) was used as a transfection reagent, according to the manufacturer’s instructions. The cells were transfected with mirVana^©^ miRNA inhibitor (Anti-miR-155-5p) miRBase accession #MIMAT0000646 (Ambion, life technologies, TX, United States) at final oligonucleotide concentration of 30 nM or mock transfection control (Lipfectamine RNAiMAX + PBS), for 24 h. After the first 24 h, transfection was stopped by changing media. ASC52telo cells were harvested for RNA extraction at 24 and 72 h to evaluate the expression of the downstream genes.

### 2.8 Gene expression analysis

Quantitative real-time PCR was performed to detect the expression of the downstream miRNA-gene targets at 24 and 72 h post-transfection (*n* = 3). Total cellular RNA was extracted using RNeasy mini kit (Qiagen, Germany). RNA was reverse transcribed into cDNA using Maxima First Strand cDNA Synthesis Kit (Thermo Fisher Scientific, United States) following the elimination of double-stranded DNA as recommended by the manufacturer. Gene expression was determined by the PowerUp^©^ SYBR green master mix (Applied biosystem, United States). Sequences for the oligonucleotide primers of target genes are listed in (Supplementary Table S1). Real-time qPCR was carried out using Applied Biosystems™ 7500 Real-Time PCR System (Applied biosystem, United States). The relative fold change in mRNA expression was calculated using the 2^−ΔΔCT^ method and normalized to the endogenous housekeeping gene Glyceraldehyde 3-phosphate Dehydrogenase (GAPDH).

### 2.9 Statistical analysis

For qPCR and protein arrays, data were analysed using the Data Analysis ToolPak (Microsoft^®^ Excel, Microsoft^®^ Office 365) and the graphs were created using GraphPad Prism Version 9 (GraphPad Software Inc., San Diego, CA). Statistical significance was evaluated using Student’s *t*-test and statistical significance was determined if *p*-value < 0.05. Bar charts showed the mathematical mean and the standard error of mean. To estimate the upregulated miRNA from the microarray analysis, *p*-value < 0.05 was considered as the level of significance. To identify the enriched pathways in the analysis, Benjamin-Hochberg (BH) corrected *p*-value < 0.05 was applied.

## 3 Results

### 3.1 Porcine wound healing revealed improved epithelialization and accelerated wound closure with autologous pAD-MSCs compared to control

**FIGURE 1:**
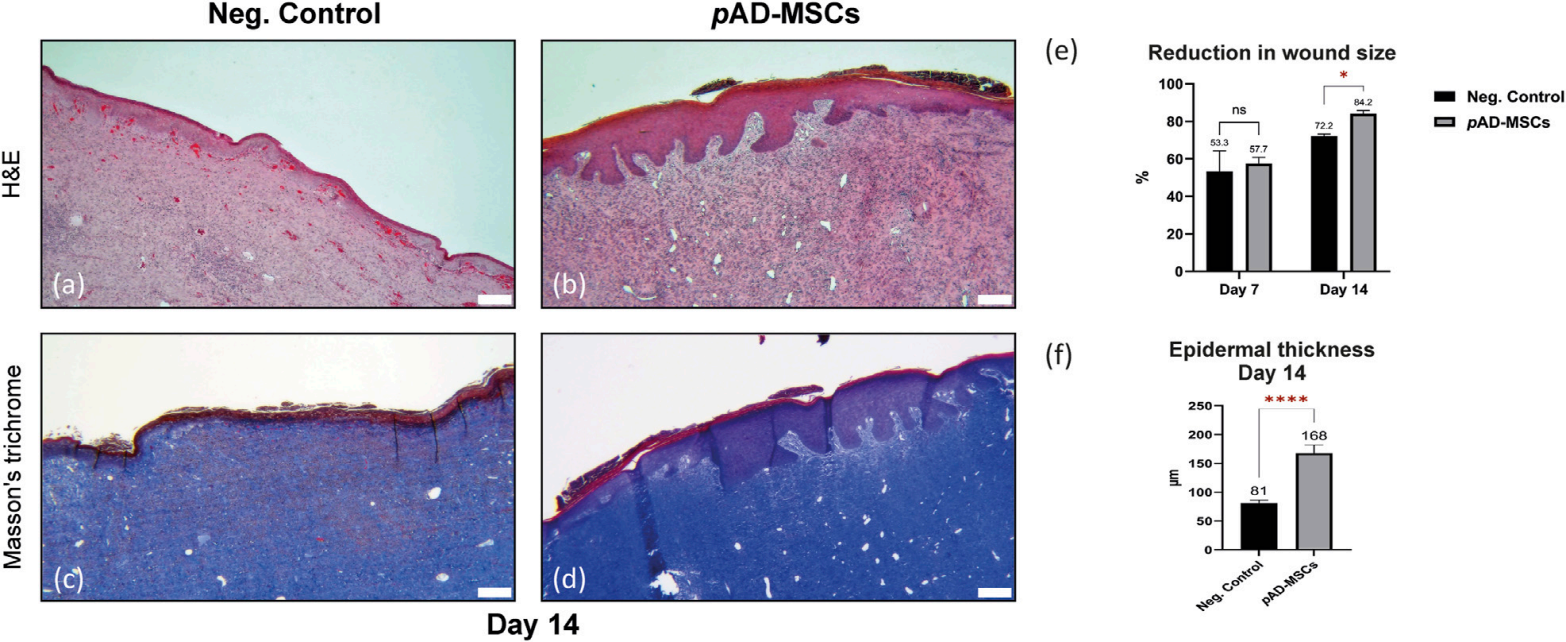
Evaluation of porcine excisional full thickness wounds at day 14. Images **(A–D)** represents the central region of the corresponding wound. **(A)** H&E staining of the cell-free control wounds exhibiting acanthotic/hyperkeratotic epidermis at the periphery, signs of purpura, blood stasis in addition to moderate-to-severe inflammatory reaction. **(B)** H&E staining of the autologous pAD-MSCs treated wounds showing uniform epithelization across the whole wound area with pronounced epidermal ridges, the dermis showing mild inflammation. **(C)** Masson’s trichrome staining of negative control showing no to little collagen disposition in negative control wounds. **(D)** Masson’s trichrome staining of the autologous AD-MSCs treated wounds showing better collagen production and arrangement in comparison to the control. **(E)** Bar chart showing the reduction in wound surface area at day 14 compared to day 0, while day 7 showing no difference. Values presented as mean percentages of D7/D0 and D14/D0 with standard error of mean (SEM). **(F)** Bar chart showing the difference in epidermal thickness, the mean with SEM of–at least-twenty read-outs were obtained for each group. Scale bars = 200 um. Statistical significance was evaluated using Student’s *t*-test for unequal variance *p < 0.05, ****p < 0.0001.

Planimetric measurements showed reduction of the wound surface area at day 14 compared to those of day 0 with 84.2% healing efficiency in *p*AD-MSCs treated wounds in respect to their corresponding initial wound size. At the meantime, negative control showed only 72.1% healing efficiency. Similar difference could not be shown at day 7. Histological evaluation of the wound biopsies at day 14 showed that the epidermis of the negative control wounds showed a variation in thickness with area of ulcerated wound. Non-healing control wounds exhibited acanthotic/hyperkeratotic epidermis at the periphery. Moreover, the dermis showed signs of purpura, blood stasis in addition to moderate-to-severe inflammation. In contrast, the treatment group showed uniform epithelization across the whole wound area, in the form of wide and thick epidermal ridges with mean (SEM) thickness of 168 (14) µm compared to that of the negative control wounds 81.2 (5.1) µm. Some parts of the epidermis of the treatment group appeared flattened in the middle, with sporadic acanthosis or hyperkeratosis. The dermis of the treatment group showed mild inflammation and fibrosis with better production and arrangement of the collagen in comparison to the control (Figure 1).

**FIGURE 2:**
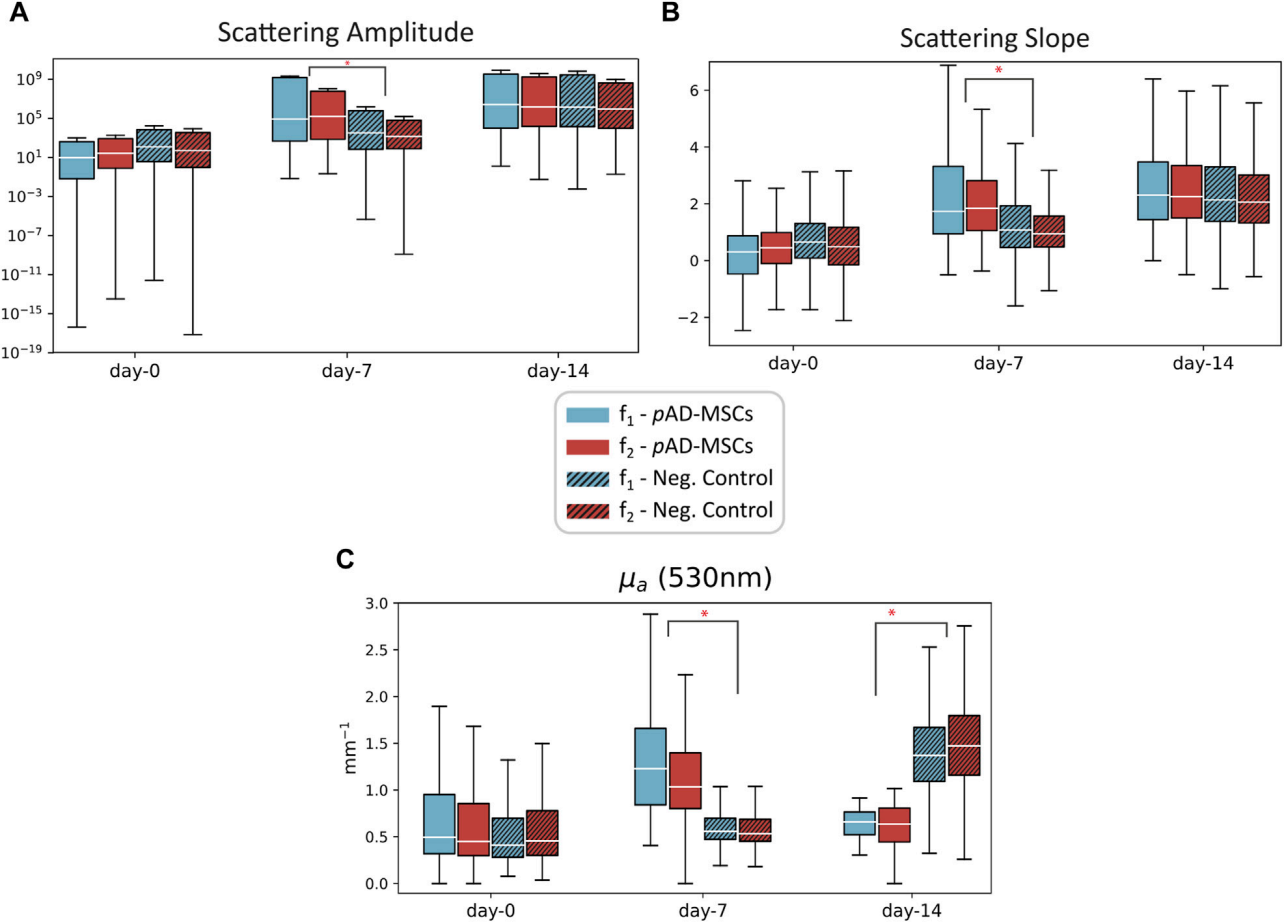
Plots of the scattering amplitude **(A)**, scattering slope **(B)** and absorption coefficient µ_a_ **(C)** measured at three time points on the porcine model on a wound treated with *p*AD-MSC (plain color), and negative control wounds (patterned color). Data from two datasets with different spatial frequencies are reported: f_1_ = [0.05–0.2] mm^−1^ (blue) and f_2_ = [0.1–0.25] mm^−1^ (red). Low spatial frequencies have higher penetration depth, so the data from f_1_ comes from deeper tissue compared to f_2_. Statistical significance was evaluated using Student’s *t*-test for unequal variance between *p*AD-MSCs and the control case *p < 0.05.

SFDI data was acquired on three wounds (two negative controls and one treated with *p*AD-MSC) at three different time points: day 0, day 7 and day 14. It was not possible to obtain SFDI data from the other *p*AD-MSC treated wound, according to the animal welfare policy. The second pig was under anaesthesia and the procedure needed more time, which could not be granted. Thus, data collection was not possible for some of the wounds. The investigators were blinded to the treatment received by each wound. The spatial frequency ranges of the datasets are f_1_ = [0.05–0.2] mm^−1^ and f_2_ = [0.1–0.25] mm^−1^. The raw data was processed and the optical parameters µ_a_ and µ’_s_ were obtained. In (Figure 2), the progression of the scattering parameters in time for the three wounds can be observed. For *p*AD-MSCs treated wound, an increasing trend in both the A and B parameter was observed, which is consistent with the formation of new tissue (new epithelial cells and ECM). In the control wounds we see a similar trend, but on a smaller scale for both A and B parameters. The difference in scattering parameters at day 7 between *pAD-MSCs* and control is statistically significant (*p* < 0.05), while their values are not significantly different at day 14 (*p* > 0.05). This suggests a slower cell growth and tissue restructuration in comparison to the *p*AD-MSCs treated wound. Furthermore, the progression of the absorption coefficient in *p*AD-MSCs treated wound showed an initial increase in µ_a_ at day 7, followed by a decrease at day 14. The higher value of µ_a_ at f_1_ compared to f_2_ also suggests that the increase in haemoglobin content is in deeper tissue, which could be an indication of increased microcirculation. In both control wounds, there are no significant changes in absorption at day 7, which suggest weak or absent tissue response. We can however see a larger increase in µ_a_ at day 14, which could be related to an inflammatory reaction, as observed in the histopathological study.

### 3.2 Analysis of miRNA microarray and protein array data reveals differentially upregulated targets in AD-MSCs

**FIGURE 3:**
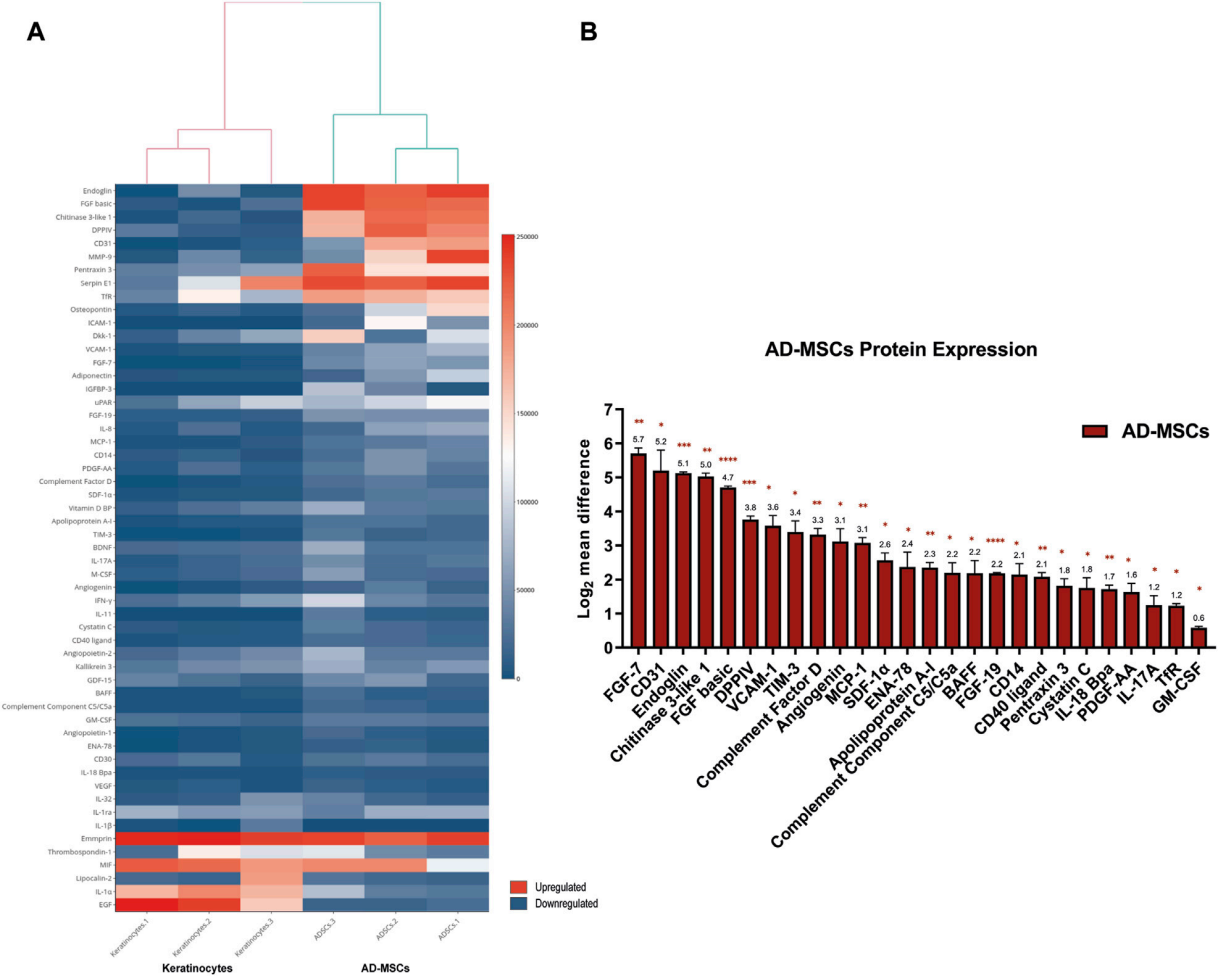
**(A)** Heatmap diagram showing the signal intensities of the detected proteins in the proteome profiler array. Heatmap visualization was created using shinyHeatmaply. The colour bar represents individual signal intensity of each sample blue (low intensity) to red (high intensity). **(B)** Bar chart detailing the 26 significantly upregulated proteins in AD-MSCs. Data represented as log_2_ mean difference in expression between AD-MSCs and keratinocytes and the standard error of mean. Statistical significance was evaluated using Student’s *t*-test for unequal variance of 3 independent donor samples (*n* = 3). *p < 0.05, **p < 0.01, ***p < 0.001, ****p < 0.0001.

Of the total 378 miRNA differentially regulated in primary AD-MSCs, 264 miRNA (69.84%) were upregulated while 114 miRNA (30.16%) were downregulated. On the other hand, proteome profiler arrays showed that 26 of the 105 soluble proteins blotted on the array membrane were significantly upregulated in AD-MSCs. Of those, the most upregulated were Fibroblast growth factor-7 (FGF-7), also known as (a.k.a) Keratinocyte growth factor (KGF) [log_2_ mean difference (MD) = 5.7, *p*-value = 0.006], CD31 a.k.a Platelet and endothelial cell adhesion molecule 1 (PECAM-1; log_2_MD = 5.2, *p*-value = 0.04), Endoglin (log_2_MD = 5.1, *p*-value = 0.0009), Chitinase 3 like 1 (log_2_MD = 5.03, *p*-value = 0.001) and Fibroblast growth factor basic (FGF-basic; log_2_MD = 4.7, *p*-value = 9.9E-06) (Figure 3; Supplementary Table S2).

### 3.3 miRNA-mRNA interactome and enrichment analysis of the miRNA-regulated targets in AD-MSCs reveal cell signalling-associated pathways

**FIGURE 4:**
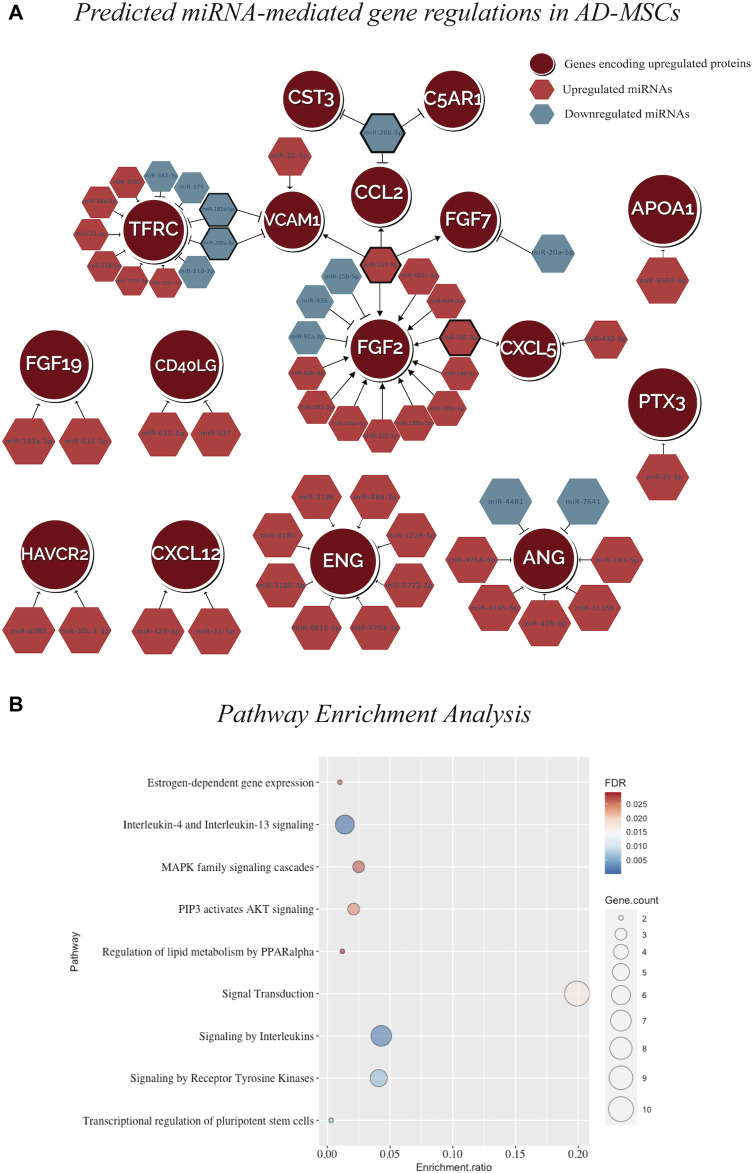
**(A)** miRNA-mRNA interaction network in AD-MSCs illustrating the predicted regulations between 54 DEmiRNAs and the genes encoding for 16 upregulated proteins. **(B)** The dot plot representing the pathway enrichment result using the GSEA algorithm. The size of the circle shows the number of genes in the pathway. The color scale represents blue (low FDR) to red (high FDR). The enrichment ratio calculates the enrichment for each pathway. The dot plot visualizing the matching 9 significantly enriched pathways by the input gene sets representing target genes of the upregulated miRNAs and genes encoding for upregulated proteins.

The fisher’s exact test (*p-*value = 0.008, two-tailed) supported the association between the 378 DEmiRNA and 28 DEproteins (Supplementary Table S3). Then the integrated analysis carried out on the 26 upregulated proteins in AD-MSCs and their corresponding protein-coding genes revealed that only 16 (of 26) matched with the known gene targets of 54 DEmiRNA (Figure 4A; Supplementary Table S3). A total of 95 and 92 significantly enriched pathways were observed resulting from the input gene lists 1 and 2, respectively (Supplementary Tables S4, S5) (Supplementary Figures S1A, B). Additionally, comparing the above results, 9 significant pathways were observed, including MAPK family signalling cascades, Transcriptional regulation of pluripotent stem cells, Signalling by Receptor Tyrosine Kinases, Signal transduction by Interleukins (Supplementary Table S6; Figure 4B; Supplementary Figure S1C).

### 3.4 Silencing of miR-155-5p validated the predicted miRNA-mediated gene regulation

**FIGURE 5:**
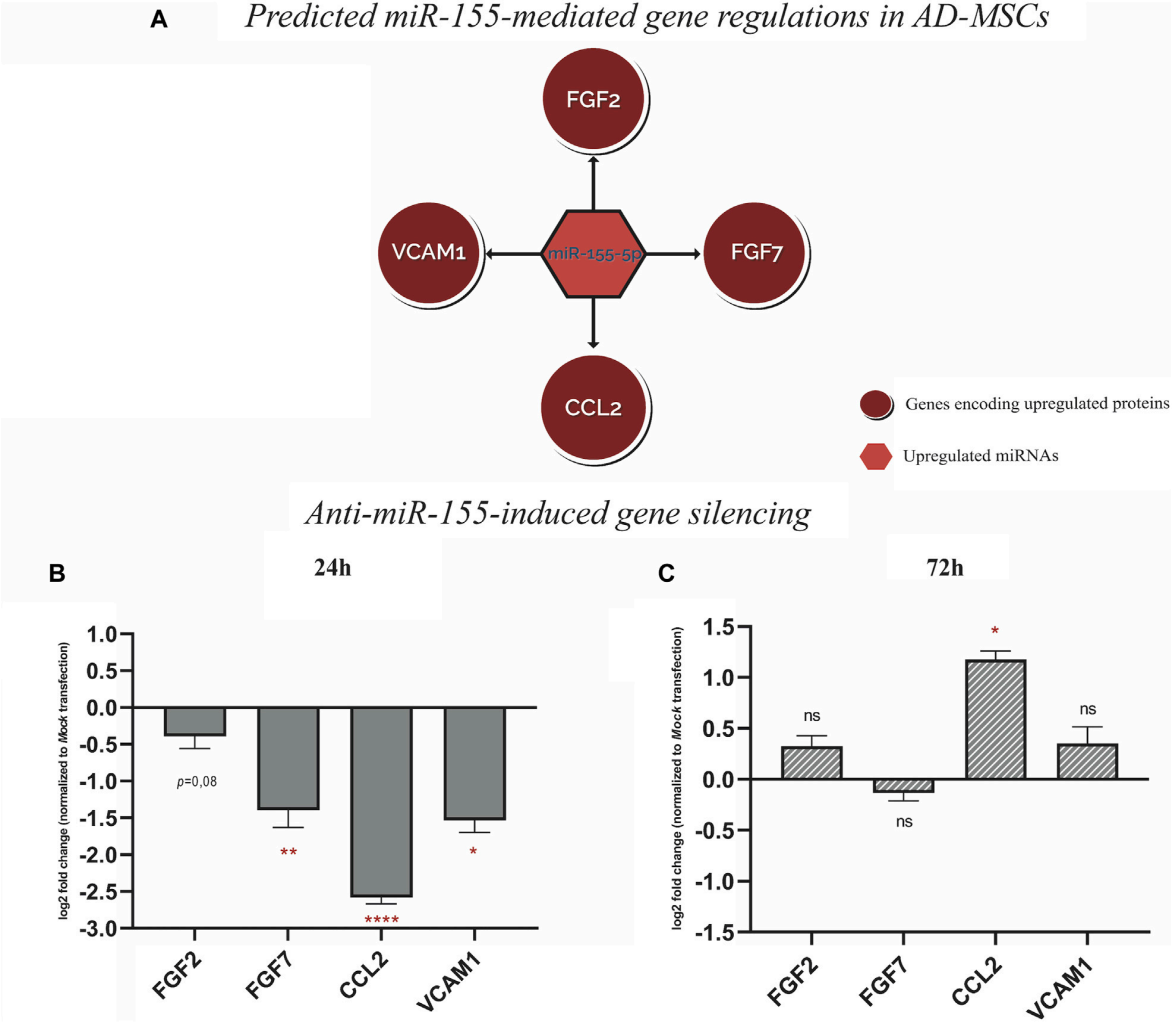
**(A)** Schematic represtentation of the predictive miR-155-mediated gene regulation with FGF2, FGF7, CCL2, and VCAM1 pretaining to wound repair. **(B)** Gene expression analysis, 24 h post-transfection with Anti-miR-155-5p showing gene silencing of FGF2, FGF7, CCL2, and VCAM1. **(C)** Gene expression analysis, showing gene silencing effect on FGF2, FGF7, CCL2, and VCAM1 alleviated 72 h post-transfection. Values in bar chart represent log2 fold change compared to mock transfection. Statistical significance was evaluated using Student’s *t*-test for unequal variance of 3 independent experiments (*n* = 3). *p < 0.05, **p < 0.01 and ****p < 0.0001.

To validate the predicted miRNA-mRNA interactome model, the positive regulation between miR-155-5p and 4 protein coding genes, which are FGF2, FGF7, CCL2 and VCAM1 was tested. These genes correspond to the upregulated proteins: FGF basic log_2_ fold change (FC) = 4.7, *p*-value = 9.9E-06), FGF-7 (log_2_FC = 5.7, *p*-value = 0.006), Monocyte chemotactic protein-1, encoded by CCL2, (MCP-1; log_2_FC = 3.1, *p*-value = 0.003) and Vascular cell adhesion molecule-1 (VCAM-1; log_2_FC = 3.6, *p*-value = 0.02) (Figure 5A). To test this hypothesis, a gene silencing experiment was conducted using miR-155-5p inhibitor. The temporal downstream effect on the predicted target genes was evaluated at 24 h (Figure 5B) and 72 h (Figure 5C) post-transfection. Gene expression analysis revealed downregulation at 24 h of FGF7 (log_2_FC = −1.4, *p*-value = 0.006), CCL2 (log_2_FC = −2.6, *p*-value = 1.7E-05) and VCAM1 at (log_2_FC = −1.5, *p*-value = 0.01), with a similar trend for FGF2 (log_2_FC = −0.4, *p*-value = 0.08) when compared to the mock transfection control without reaching statistical significance (Figure 5B). The inhibitory effect of miR-155-5p appeared to be only transient as the majority of the genes mitigated at 24 h, reverted to their baseline expression levels after 72 h from terminating the transfection and alleviating the gene-silencing effect FGF2 (log_2_FC = 0.3, *p*-value = 0.07), FGF7 (log_2_FC = −0.1, *p*-value = 0.1), VCAM1 (log_2_FC = 0.4, *p*-value = 0.2). However, CCL2 appeared to have been upregulated following the transient downregulation as it showed a surge of expression at log_2_FC = 1.2, *p*-value = 0.01) (Figure 5C).

## 4 Discussion

The role of AD-MSCs in wound healing is attracting more attention due to their feasibility and efficacy. This study verified the regenerative capacity of AD-MSCs auto-transplants in porcine full-thickness excisional wounds. At day 14, histological and morphometric analyses showed AD-MSC expedited wound closure compared to naturally healed wounds. Similar findings were reported when the cells were applied to a burn wound as subdermal injection or topical spray (Foubert et al., 2016). Our findings were verified by histopathology, as well as by SFDI. The latter can be considered as a non-invasive technique, which can be directly correlated to biological markers normally associated with wound healing. The absorption coefficient (µ_a_) gives an indication of the concentration of chemical species in tissue that are able to absorb photons. Molecules that absorb light within the spectral range of the imaging system used in this investigation are haemoglobin and melanin (Jacques, 2013). The latter was excluded from the analysis as the epidermis was surgically removed during the wound creation step. Thus, µ_a_ at ∼530 nm was selected to represent the spatially resolved total haemoglobin present within the wound area (Saager et al., 2018). This parameter gives an approximate indication of the quantity of blood present in the tissue (e.g., from micro-circulation or haemoglobin degradation products). In stem-cells treated wound, the scattering parameters suggested a larger degree of cell repopulation, while the absorption (from haemoglobin) showed a faster reactivity of the tissue in time, when compared to negative control. An important contribution of these optical measurements is the introduction of additional time points during the healing process. This complements the data obtained from the biopsy, which can only be obtained at the end of the study, allowing a more comprehensive assessment in time. However, a low number of wounds were obtained from the current porcine wound model, which represents a limitation of this study. Future studies should include more animals or more wounds per animal, to further confirm our findings.

The next step of the study was to investigate the molecular signalling cascades associated with AD-MSCs’ effect in wound healing. This study analysed the previously identified miRNA expression profile in AD-MSCs (Shahin et al., 2023); integrated with the identified proteome signature of AD-MSCs. This integration enabled us to generate a predicted positive regulation between miR-155 and genes encoding for FGF2, FGF7, CCL2, and VCAM1 in human AD-MSCs. The inhibition of miR-155 elicited gene silencing for FGF2, FGF7, CCL2, and VCAM1 for 24 h, validating the predicted miR-155-mediated gene regulation. The positive regulation role of miRNA on target genes can have multiple mechanisms, including and not limited to, 1) miRNA-mediated post-transcriptional upregulation 2) translation upregulation or 3) competition with repressive proteins preventing them from binding to their target sites, which increases mRNA stability and promotes the expression of target protein. Nevertheless, miRNA-mediated regulation of gene expression is a reversible action (Valinezhad Orang et al., 2014). Thus, a single miRNA can exert both positive and negative gene regulations, and similarly a single gene could respond in both modulations depending on system-specific conditions.

miR-155 has been recognized as a regulator and respondent to multiple inflammatory mediators involved in the cellular immune response against pathogens including interleukins, tumor necrosis factor and interferons (Mahesh and Biswas, 2019; Rodriguez et al., 2007; O’Connell et al., 2007). Silencing of miR-155-5p in atopic dermatitis mouse model reduced both the thickening of the epidermis and the expression of T helper type 2 (Th2) immune response-associated cytokines, thymic stromal lymphopoietin and interleukin-33, attenuating the overall allergic inflammation (Wang et al., 2019). miR-155 is among the first miRNA to be associated with the ability of inflammatory cells to recognize invading pathogens particularly through Toll-like receptors (TLRs). Thus, miR-155 can have an important role in wound healing research, particularly in chronic infected wounds (Martinez-Nunez et al., 2009). Furthermore, miR-155 is associated with acceleration of epithelial cell migration through promoting TGF-β-induced epithelial-to-mesenchymal transition and tight junction dissolution in normal murine mammary gland epithelial cells (Kong et al., 2008). The role of miR-155 in wound healing was elucidated in a full-thickness excision wounds in rats and shown to have accelerated cutaneous wound closure independent of wound contraction. The authors provided *in-vitro* evidence that the regenerative effect of overexpressing miR-155 was attributed to enhanced keratinocytes migration via the activation extracellular proteins, particularly matrix metalloproteinase-2 (MMP-2) (Yang et al., 2017). In mesenchymal stem cells isolated from aged bone marrow donors, miR-155 was found to be considerably higher than that of young donors MSCs. Additionally, upregulation of miR-155 in young MSCs led to increased cellular senescence signified by increased signal of senescence-associated β-galactosidase (SA-β-gal). This proposes a positive correlation of miR-155 expression with cellular senescence of MSCs through regulating the Cab39/AMPK signalling pathway. Aged MSCs can help to improve the cardiac function when miR-155 was inhibited in the cells prior to the injection of the infarcted myocardium in murine model. The effect was evident by enhanced angiogenesis and cell survival in addition to reduced infract size as well as cardiomyocyte apoptosis (Hong et al., 2020). Beside cellular senescence, there is scarcity in the literature describing the role of miR-155 in AD-MSCs. In addition, miR-155 is released as a main constituent of the exosomal miRNA cargo in adipose tissue macrophages (ATMs) which can be transferred to and modulate insulin sensitivity in other cell types such as adipocytes, myotubes or hepatocytes (Ying et al., 2017). Furthermore, miR-155 was detected in the extracellular vesicles isolated from AD-MSCs (Constantin et al., 2022). In intervertebral disc degeneration (IDD), AD-MSCs-derived exosomal miR-155-5p showed to inhibit pyroptosis and promoted autophagy and ECM synthesis in nucleus pulposus cells, *in-vitro,* through targeting TGF beta receptor 2 (TGFβR2). In another model, exosomes were isolated form AD-MSCs overexpressing miR-155 and injected in IDD rats which was associated with improved symptoms of IDD in relation to enhanced autophagy and reduced pyroptosis (Chen et al., 2023). The histopathological evaluation of our porcine wounds showed less inflammation in the wounds receiving *p*AD-MSCs. The potential of miR-155 to mitigate the inflammatory response and elicit a positive regenerative effect in the early phases of wound repair constitute an interesting premise to explore in the future.

The proteome screening showed upregulation of FGF2, FGF7 and FGF19 in AD-MSCs. These growth factors play a crucial role in various regenerative processes, including cell proliferation, differentiation and migration, as well as mediating angiogenesis (Maddaluno et al., 2017). According to experimental target verification, both FGF2 and FGF7 are positively regulated by miR-155-5p in AD-MSCs. The positive regulation role of miR-155 was previously reported in B cells and associated with specific antibodies production, as well as enhanced calcium accumulation in response to anti-IgM antibody. The suggested mechanism was through the inhibition of SHIP1, a tyrosine kinase negative regulator. In addition, miR-155 has been shown to enhance TNF-α and IL-6 in macrophages (Mashima, 2015). FGF2 was among the most abundant angiogenesis inducers detected in mature human adipose tissue extracts as well in AD-MSCs-released exosomes (Sarkanen et al., 2012; Kim et al., 2017). FGF2 is also recognized as a key supplement in AD-MSCs culture medium as FGF2 enhances proliferation and maintain the cell stemness. FGF2 depletion in serial AD-MSCs cultivation has been shown to induce autophagy and senescence while suppressing stemness genes (Ma et al., 2019; Cheng et al., 2020). In the same context, FGF7 was also upregulated in AD-MSCs and was shown to be a direct target for miR-155-5p. FGF7 is generally recognized as keratinocyte growth factor and was found to be abundant among the soluble paracrine factors detected in adipose tissue, as it is normally produced by cells of mesenchymal origin (Gabrielsson et al., 2002). Ceccarelli et al. (2018) demonstrated that human AD-MSCs showed elevated expression of FGF7 during early phases of adipogenic differentiation. In keratinocytes, FGF7 is a major regulator that promotes Tumor Necrosis Factor Alpha (TNF-α) in human keratinocytes through the FGFR2–AKT–NF–κB signalling axis. FGF7 targets epithelial cells and exerts an important role in modulating cellular processes such as proliferation and migration, in addition to vasculogenesis, and regeneration of various organs (Ceccarelli et al., 2018). Primarily secreted by mesenchymal cells, FGF7 exerts its effect through paracrine signalling on epidermal keratinocytes but is not necessarily secreted by them. FGF7 binds to the KGFR2IIIb receptor found exclusively on keratinocytes, and promotes the migration, proliferation and differentiation of epidermal keratinocytes triggering the inflammatory cascade during wound healing (Belleudi et al., 2010; Pastar et al., 2014).

Both FGF2 and FGF7 are crucial factors for AD-MSCs to exhibit their regenerative properties in wound healing, enabling epithelial tissues to restore structure and function. Both have been reported to be implicated in the early and late stages of wound healing including formation of granulation tissue, re-epithelialization as well as the remodelling phase of wound healing (Werner et al., 1994; Demidova-Rice et al., 2012; Prudovsky, 2021). FGF2 has important roles in many cells including dermal fibroblasts, keratinocytes, endothelial cells, and melanocytes. FGF2 stimulates angiogenesis and enhances the production of ECM proteins, which are essential for tissue repair in the formation of granulation tissue. The mitogenic and angiogenic properties of FGF2 enable its role in tissue remodelling and neovascularization (Lee et al., 2021). FGF2 is known to induce neoangiogenesis during tissue repair, tissue engineering and wound healing through stimulating the differentiation of AD-MSCs into endothelial cells (Mazini et al., 2020), or through enhancing the ingrowth of blood vessels by directly incorporating FGF2 in bioengineered scaffolds (Shahin et al., 2020). At the wound site, the remodelling effect of FGF2 takes place as a result of efficient inhibition of fibroblast terminal differentiation to myofibroblast, which is a key mediator when it is activated in keloids and hypertrophic scars (Akita et al., 2013).

Another significantly upregulated protein in AD-MSCs and validated target for miR-155-5p, is the proinflammatory cytokine Chemokine (CC-motif) ligand 2 (CCL2) a.k.a Monocyte chemotactic protein-1 (MCP1). In immune response, CCL2 promotes inflammation by binding to its receptors C-C chemokine receptor type 2 (CCR2) and subsequently activating monocytes recruitment as well as mediating neutrophil and macrophage infiltration (Khan et al., 2013; Singh et al., 2021). A recent study reported that addition of CCL2 directly promoted adipogenesis by enhancing lipid accumulation in AD-MSCs as well as accelerated angiogenesis by stimulating tube formation of Human Umbilical Vein Endothelial Cells (HUVECs) in cell culture. The authors argue that the latter effect is believed to be attributed to CCL2 upregulating the expression of key vascularization genes VEGF and VEGFR2 as well as MMPs through the PI3K‐AKTpathway (Zhu et al., 2022). Conversely, from a wound healing perspective, CCL2 plays a major role in its earlier phases as it modulates T cell differentiation toward the Th2 subset (Dipietro et al., 2001). It was among the earliest and most upregulated genes during the acute inflammatory phase of murine excisional wounds screened at 6–12 h post wounding, with an order of ∼70 fold-change (Roy et al., 2008). Recent evidence highlighted the importance of Nuclear factor erythroid 2 (Nrf2) together with CCL2 in the healing of epidermal defects in diabetic mice. The study unveiled an immunogenic signalling network centred on epidermal keratinocyte-macrophage crosstalk, via Nrf2/CCl2/EGF signalling axis. Nrf2 promotes CCL2 expression to mediate macrophage trafficking and direct macrophage production of epidermal growth factor (EGF), which in turn activates the epidermal progenitors triggering an early regenerative response after injury (Villarreal-Ponce et al., 2020). In another study, CCL2 showed discernible effect in treating early-stage diabetic wounds as it stimulated the healing by accelerating immune cell infiltration and restoring the macrophage response. Inflammation subsided earlier in CCL2-treated wounds, alleviating the otherwise persistent, hyper-inflammatory mode characteristic of diabetic wounds in 10 days (Wood et al., 2014).

VCAM1 is an endothelial adhesion molecule to constitute the 4th arm of the proposed miR-155 mediated gene regulation in AD-MSCs. Otherwise known as CD106 is a cell adhesion factor known to play a role in regulating stem cell trafficking by modulating the homing or mobilization of stem cells. Unlike in bone marrow-derived stem cells, the pattern of AD-MSCs expressing VCAM1 is preserved (Madonna et al., 2009). Monocytes and lymphocytes can adhere to VCAM1 allowing them to tag along, penetrating the endothelial surface and inducing a circulating inflammatory response (Yi et al., 2020). VCAM1 expression was found to be overexpressed at the wound edge at 48–96 h post injury in murine excisional wounds (Roy et al., 2008). The pro-angiogenic effect of VCAM1 signalling was evident in bone defect repair when treated with human serum-derived exosomes as VCAM1 positively regulates vascular endothelial growth factor (VEGF) (Xiang et al., 2023). In our experimental target analysis, the reported gene silencing effect of Anti-miR-155 on FGF2, FGF7, CCL2 and VCAM1 reverted to the basal level at 72 h as the method used for introducing miRNA to the cells was based on cationic-lipid transfection. For obtaining stable transfection, DNA integration of miR-155-5p loaded plasmid could have been used. However, transient transfection was sufficient to prove the effect of miR-155-5p on the proposed targets.

The rest of the upregulated panel in AD-MSCs can be grouped into cytokines, growth factors, and chemokines, which can be involved in inflammatory and vasculogenic processes. Both are closely intertwined processes in the context of wound healing. Inflammatory cells often release angiogenic factors, thereby exerting mitogenic and migratory effects on the endothelium (Yi et al., 2020). The list of upregulated inflammatory mediators include: Pentraxin-related protein (PTX3), Complement 5a anaphylatoxin chemotactic receptor 1 (C5AR1), C-X-C motif chemokine ligand 5 (CXCL5), CD40 ligand (CD40LG), C-X-C Motif Chemokine Ligand 12 (CXCL12), Hepatitis A Virus Cellular Receptor 2 (TIM-3/HAVCR2) and Transferrin receptor protein 1 (TFRC). The list of upregulated proangiogenic factors and vasculogenic cytokines beside FGFs and VCAM1 include: Angiogenin (ANG), platelet and endothelial cell adhesion molecule 1 (PECAM1/CD31) and Endoglin (ENG/CD105). These findings are in agreement with the well-known immunomodulatory role of AD-MSCs *in-vivo* (Mazini et al., 2020).

Furthermore, our pathway enrichment analysis highlighted the signal transduction, inflammatory and immune response pathways. As inflammatory cytokines, interleukins (IL)-1, 6, 8, and 17 are among the initial factors to be produced in response to skin wounds in order to participate in the inflammatory phase of wound healing (Xiao et al., 2020). IL-8, among other cytokines, attracts inflammatory polymorphonuclear cells (PMNs) in large numbers within the first 24–48 h at the wound site. PMNs in turn act as a major producer of proinflammatory cytokines, including IL-1α, IL-1β, IL-6, and TNF-α. 2–3 days post injury, monocytes are recruited to the wound bed and remain there for weeks. Monocytes transform into macrophages, and secrete IL-1α, IL-1β, IL-6, and TNF-α to perpetuate the inflammatory state. This inflammatory phase persists for the first 4 days in normal wound healing and is crucial to initiate downstream repair and mediate angiogenesis (Kanji and Das, 2017). Although the cytokines produced by AD-MSCs are involved in the inflammatory signalling cascades, the established immunomodulatory role of exogenous AD-MSCs in wound repair is anti-inflammatory in nature. AD-MSCs can downregulate the immune response through 3 main ways 1) secreting immunosuppressive factors such as IL-10 and transforming growth factor-beta (TGF-β) which can promote maturation of suppressor T cells, 2) reducing dendritic cell maturation, and 3) inhibiting the proliferation of natural killer (NK) cells (Kokai et al., 2014; Zhou et al., 2022). This immunosuppressive feature promotes AD-MSCs as suitable candidates for allogenic cell transplantations. AD-MSCs attenuate wound inflammation by inhibiting the acute immune response of the host and inducing healing, even in chronic inflammatory state. MAPK family signalling cascade is another signalling pathway upregulated by AD-MSCs according to our pathway enrichment analysis. P38/MAPK promotes ECM production via positively regulating collagen synthesis (Du et al., 2013). It would be interesting to verify if the involvement of AD-MSCs in wound repair is essential through differentiation into keratinocytes, or that AD-MSCs secretions are sufficient to enhance the natural repair mechanisms.

miRNA-based therapeutics are emerging as promising strategy with the potential to improve wound healing. Efforts being devised in preclinical studies in order to overcome the challenges associated with miRNA therapies exemplified in guiding sufficient dosing and developing efficient delivery methods that maintain miRNA stability in the wound environment (Banerjee and Sen, 2015; Veith et al., 2019). The synthesis of the double stranded miRNA mimics enhanced their stability as well as their potential for clinical use. To avoid degradation and enhance targeting, miRNA can be conjugated with lipid particles or loaded into specific viruses or nanoparticles (Ho et al., 2022). Skin wounds have the advantage of being accessible. The strategy of applying miRNA in an ointment, as a cell-free therapy, can be feasible for patients, decrease the incidence of non-target effects and allow to identify local side effects (Inoue et al., 2020). Furthermore, pre-treatment of stem cells with epigenetic modulators can enhance their response to the differentiation conditions (El-Serafi and Hayat, 2012). It would be interesting to investigate the effect of transient transfection with miR-155 on stem cell differentiation efficiency into keratinocyte-like cells. These cells can be transplanted at the wound as miR-155-primed cell-based therapy.

## 5 Conclusion

This study provides *in-vivo* evidence that corroborates the restorative effect of AD-MSCs in full-thickness excisional wounds. AD-MSCs accelerated wound closure and achieved satisfactory epithelization in 14 days compared to naturally healed wounds as shown with histological, morphometric and SDFI evaluation. The latter can be considered as a non-invasive technique that provides read-outs indicative of tissue repair during the healing process. Future studies should validate possible correlations between SFDI readings, ideally with a higher number of wounds. Our computational analysis of the differentially expressed miRNAs and proteins in human AD-MSCs and keratinocytes predicted that miR-155 may potentiate the immunomodulatory effect of AD-MSCs by positively regulating the key proteins FGF2, FGF7, CCL2, and VCAM1. Furthermore, the predicted regulation was validated experimentally through transfection with miR-155 inhibitor and confirmed a positive regulation between miR-155 and the identified four factors. Each of these factors carries out key functions at different events within the wound healing process including vascularization, inflammation, proliferation, and remodelling.

As a follow up study, the therapeutic potential of miR-155 or AD-MSCs overexpressing miR-155 should be investigated *in-vivo*, particularly in difficult-to-heal wound models. However, two critical aspects about miR-155 must be taken into consideration: firstly, to elucidate its immunomodulatory effect in order to eliminate the risk of exacerbating the inflammatory state at the wound site. Secondly, the underlying mechanisms implicating miR-155 expression with cellular senescence reported in the context of aged mesenchymal stem cells. Future work should explore the potential feedforward/back loops affecting how miR-155 regulates the expression of FGF2, FGF7, CCL2, and VCAM1 and how these proteins interact with one another, in addition to the potential reciprocal effects of these proteins on miR-155 expression. Lastly, the intermediary proteins potentially underlying the positive regulation between miR-155 and the identified proteins have not been studied in this report and constitute a limitation of this study.

## Data Availability

The original contributions presented in the study are included in the article/Supplementary Material. Further inquiries can be directed to the corresponding author.

## Supplementary material

SUPPLEMENTARY FIGURE S1
**(A,B)** The dot plot describing the pathway enrichment result using the GSEA algorithm. Gene count is the number of genes found in the database from the user data on the pathway. The size of the circle shows the number of genes in the pathway. The color scale represents blue (low FDR) to red (high FDR). The enrichment ratio calculates the enrichment for each pathway. **(C)** The Venn diagram shows the overlapping number of pathways.
