# Supplementary material for: MicroRNA-155 mediates multiple gene regulations pertinent to the role of human adipose-derived mesenchymal stem cells in skin regeneration
Source: Front Bioeng Biotechnol. 2024 Mar 18;12:1328504. doi: 10.3389/fbioe.2024.1328504 (PMC10982420; doi:10.3389/fbioe.2024.1328504)
Supplement: Supplementary file 3 [file Table1.DOCX]

Supplementary Material

MicroRNA-155 mediates multiple gene regulations pertinent to the role of human adipose-derived mesenchymal stem cells in skin regeneration

**Hady Shahin ^1,2,3^, Luigi Belcastro ^4^, Jyotirmoy Das ^5,6^, Marina Perdiki Grigoriadi ^7^, Rolf B Saager ^4^, Ingrid Steinvall ^1^, Folke Sjöberg ^2^, Pia Olofsson ^1^, Moustafa Elmasry ^1^ and ^*^Ahmed T El-Serafi ^1,2,*^**

*** Correspondence:** Ahmed El-Serafi: ahmed.elserafy@liu.se

**Supplementary Table 1. Oligonucleotides primers used in the study.**

| **Name** | **Forward Primer** | **Reverse Primer** | **Accession#** | **Reference** |
| --- | --- | --- | --- | --- |
| ***GAPDH*** | CCTGCACCACCAACTGCTTA | GGCCATCCACAGTCTTCTGAG | >NM_001357943.2 | ^1^ |
| ***FGF2*** | TGTGCTAACCGTTACCTGGCT | CAGTGCCACATACCAACTG | >NM_002006.6 | ^2^ |
| ***FGF7*** | TGGCAATCAAAGGGGTGGAA | GCCATAGGAAGAAAGTGGGCT | >NM_002009.4 | ^3^ |
| ***VCAM1*** | GTCTCCAATCTGAGCAGCAA | TGGGAAAAACAGAAAAGAGGTG | >NM_001078.4 | ^4^ |
| ***CCL2*** | AGGTGACTGGGGCATTGAT | GCCTCCAGCATGAAAGTCTC | >NM_002982.4 | ^5^ |

Abbreviations: **GAPDH** (Glyceraldehyde 3-phosphate Dehydrogenase), **FGF2** (Basic fibroblast growth factor), **FGF7** (Fibroblast growth factor 7 / Keratinocyte growth factor), **VCAM1** (Vascular cell adhesion molecule 1 / CD106), **CCL2** (Chemokine C‐C motif ligand2).

**References**

1. Zhang Y, Roos M, Himburg H, et al. PTPsigma inhibitors promote hematopoietic stem cell regeneration. *Nat Commun*. Aug 14 2019;10(1):3667. doi:10.1038/s41467-019-11490-5

2. Quennell JH, Stanton JA, Hurst PR. Basic fibroblast growth factor expression in isolated small human ovarian follicles. *Mol Hum Reprod*. Sep 2004;10(9):623-8. doi:10.1093/molehr/gah083

3. Lammermann I, Terlecki-Zaniewicz L, Weinmullner R, et al. Blocking negative effects of senescence in human skin fibroblasts with a plant extract. *NPJ Aging Mech Dis*. 2018;4:4. doi:10.1038/s41514-018-0023-5

4. Zhang D, Bi J, Liang Q, et al. VCAM1 Promotes Tumor Cell Invasion and Metastasis by Inducing EMT and Transendothelial Migration in Colorectal Cancer. *Front Oncol*. 2020;10:1066. doi:10.3389/fonc.2020.01066

5. Qian BZ, Li J, Zhang H, et al. CCL2 recruits inflammatory monocytes to facilitate breast-tumour metastasis. *Nature*. Jun 8 2011;475(7355):222-5. doi:10.1038/nature10138
