# Supplementary figures and images for: MicroRNA-155 mediates multiple gene regulations pertinent to the role of human adipose-derived mesenchymal stem cells in skin regeneration
Source: Front Bioeng Biotechnol. 2024 Mar 18;12:1328504. doi: 10.3389/fbioe.2024.1328504 (PMC10982420; doi:10.3389/fbioe.2024.1328504)

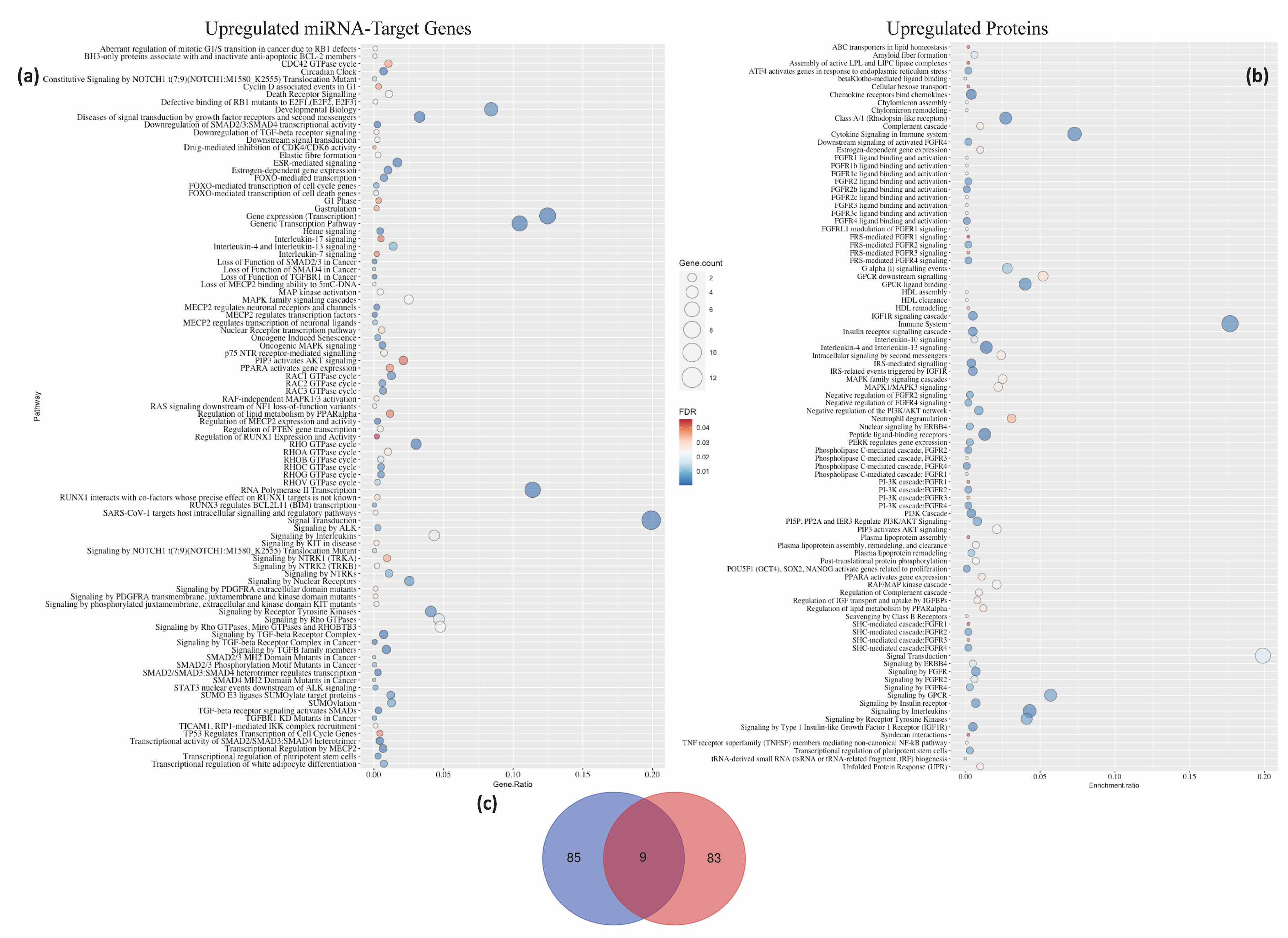

Supplement: Supplementary file 4 [file Image1.TIF]
